# Association of the metabolic score for insulin resistance with bone mineral density and trabecular bone score in cancer patients: a cross-sectional study

**DOI:** 10.3389/fendo.2026.1938781

**Published:** 2026-09-07

**Authors:** Hongdan Yao, Cheng Niu, Lin Zhu, Peiying Lin, Zefan Chen, Huling Wen, Renxian Xie

**Affiliations:** 1 Department of Nuclear Medicine, Cancer Hospital of Shantou University Medical College, Shantou, China; 2 Department of Radiation Oncology, Cancer Hospital of Shantou University Medical College, Shantou, China

**Keywords:** bone mineral density, cancer patients, insulin resistance, metabolic score for insulin resistance, trabecular bone score

## Abstract

**Objective:**

To investigate the association between the Metabolic Score for Insulin Resistance (METS-IR) and bone mineral density (BMD) and trabecular bone score (TBS) in adult cancer patients.

**Methods:**

This cross-sectional study enrolled 127 patients (median age 63 years, 44.9% female) with histopathologically confirmed malignancies. BMD at the lumbar spine, femoral neck, and total hip and lumbar spine TBS were measured by dual-energy X-ray absorptiometry. METS-IR was calculated using fasting glucose, triglycerides, high-density lipoprotein cholesterol, and body mass index. Multivariable linear regression was used to identify independent associations.

**Results:**

After adjustment for confounders, METS-IR was independently and positively associated with BMD at lumbar spine (β = 0.006, 95% CI 0.002–0.011, P = 0.007), femoral neck (β = 0.007, 95% CI 0.003–0.011, P < 0.001), and total hip (β = 0.007, 95% CI 0.003–0.010, P < 0.001). Age was inversely associated with lumbar spine and femoral neck BMD, but not with total hip BMD; bone metastasis was linked to higher lumbar spine and total hip BMD. In contrast, METS-IR showed no significant association with lumbar spine TBS (P = 0.374). For TBS, age and male sex, but not METS-IR, were independent determinants.

**Conclusions:**

In cancer patients, higher METS-IR is independently associated with greater BMD but not with better trabecular microarchitecture. This discordance between bone quantity and quality suggests that insulin resistance may mask impaired bone quality despite preserved BMD. Clinicians should consider bone quality assessments when evaluating fracture risk in metabolically compromised cancer patients. Longitudinal studies are warranted to confirm these findings.

## Introduction

Cancer patients are at high risk of bone loss and fragility fractures due to a combination of disease-related factors (such as systemic inflammation and hormonal dysregulation) and treatment-related adverse effects (such as chemotherapy, glucocorticoids, and androgen deprivation therapy) (1–4). Bone mineral density (BMD), measured by dual-energy X-ray absorptiometry (DXA), is the standard clinical tool for diagnosing osteoporosis and assessing fracture risk (5). In addition, trabecular bone score (TBS), a gray-level texture analysis derived from lumbar spine DXA images, provides an indirect assessment of trabecular microarchitecture and has been shown to independently predict fracture risk in various populations, including patients with cancer (6, 7). Consequently, investigating clinical factors that influence both BMD and TBS is essential for a comprehensive evaluation of skeletal health in oncology patients.

Insulin resistance (IR), a state of reduced cellular responsiveness to insulin, is a hallmark of metabolic syndrome and is commonly associated with obesity, chronic inflammation, and cardiovascular disease (8). Emerging evidence suggests that IR may also affect bone metabolism through multiple pathways, including hyperinsulinemia-induced anabolic effects on bone, altered secretion of adipokines and inflammatory cytokines, and accumulation of advanced glycation end-products (8). However, the relationship between IR and bone mass remains controversial: some studies report a positive association between IR and higher BMD—likely due to the osteoanabolic action of insulin and greater mechanical loading (9)—while others suggest that IR-related inflammation and collagen cross-linking may impair bone material properties and increase fracture risk (10). Most prior studies have relied on the homeostasis model assessment of insulin resistance (HOMA-IR), which requires fasting insulin measurement, limiting its application in retrospective or large-scale studies (11).

To overcome this limitation, the Metabolic Score for Insulin Resistance (METS-IR) was developed as a novel, non-insulin-based index derived from routine clinical measurements. METS-IR has been well validated against the euglycemic-hyperinsulinemic clamp and has demonstrated superior performance in identifying IR compared with other simple indices such as the Triglyceride-Glucose (TyG) index (12, 13). Because METS-IR integrates fasting glucose, lipid profiles, and body mass index, it captures a broader metabolic phenotype and is particularly suited for retrospective clinical investigations.

To date, the association between METS-IR and skeletal health in cancer patients has not been explored. Given the high prevalence of both metabolic disturbances and bone fragility in this population, we hypothesized that METS-IR might serve as an independent determinant of BMD and TBS. Therefore, the present study aimed to investigate the independent associations between METS-IR and lumbar spine BMD, femoral neck BMD, total hip BMD, and lumbar spine TBS in a cohort of adult cancer patients.

## Methods

### Study design and participants

This cross-sectional study was conducted at the Cancer Hospital of Shantou University Medical College between November 2024 and July 2025. Consecutive adult patients (aged ≥18 years) with a histopathologically confirmed diagnosis of malignant tumor were considered for inclusion. Inclusion criteria were as follows: (1) age ≥18 years; (2) Eastern Cooperative Oncology Group (ECOG) performance status 0–2; (3) an expected survival of at least 3 months as judged by the treating oncologist; (4) availability of a clinically indicated dual-energy X-ray absorptiometry (DXA) scan, with complete bone mineral density (BMD) measurements at the lumbar spine (L1–L4), femoral neck, and total hip; (5) availability of fasting venous blood samples, including fasting plasma glucose, triglycerides, high-density lipoprotein cholesterol (HDL-C), and anthropometric data required for the calculation of the Metabolic Score for Insulin Resistance (METS-IR) (body weight and height).

Exclusion criteria included: (1) lack of histopathological confirmation of malignancy; (2) absence of DXA BMD or TBS data; (3) presence of two concurrent primary cancers; and (4) missing data for any of the variables required to compute METS-IR. After applying these criteria, the final analytic sample was determined, and the flow diagram of patient selection is presented in the Results section.

### Data collection

Demographic and clinical characteristics, including age, sex, tumor type, histological subtype, presence of bone metastasis, prior fracture history, smoking status, long-term glucocorticoid use, and alcohol consumption (>3 units per day), were extracted from electronic medical records. Body weight and height were measured with participants wearing light clothing and no shoes, and body mass index (BMI) was calculated as weight in kilograms divided by height in meters squared. Blood samples were collected after an overnight fast of at least 8 hours. Fasting plasma glucose, total cholesterol, triglycerides, and HDL-C were measured using standard enzymatic methods on an automated biochemistry analyzer.

### Measurement of BMD and TBS

BMD (g/cm²) was measured at the lumbar spine (L1–L4), femoral neck, and total hip using a dual-energy X-ray absorptiometry scanner (iDXA, GE Healthcare Lunar, Madison, WI, USA). Daily quality control and calibration procedures were performed according to the manufacturer’s recommendations. All scans were analyzed by a single trained technician using standard software (enCORE version 18.0, SP 4.1). The *in vivo* short-term coefficients of variation (CV) for BMD measurements at our institution were 1.0% for the lumbar spine, 1.2% for the femoral neck, and 1.0% for the total hip, determined in accordance with the International Society for Clinical Densitometry (ISCD) guidelines.

Lumbar spine TBS was derived from the same DXA images using TBS iNsight software (version 3.0.0.15, Medimaps, Geneva, Switzerland). TBS was calculated as the mean value of the L1–L4 vertebrae, using a region of interest identical to that used for lumbar spine BMD. TBS values were not computed for vertebrae with severe structural artifacts or surgical hardware. All participants were scanned in a fasting state, wearing light clothing without metal fasteners. These data were used for descriptive analyses.

### Calculation of METS-IR

METS-IR was calculated using the following formula, with glucose and triglycerides originally measured in mmol/L converted to mg/dL prior to computation:

METS-IR = ln[(2 × Glucose [mg/dL]) + Triglycerides [mg/dL]] × BMI [kg/m²]/ln[HDL-C [mg/dL]]
Conversion factors were: glucose (mmol/L) × 18.02 = glucose (mg/dL); triglycerides (mmol/L) × 88.57 = triglycerides (mg/dL); HDL-C (mmol/L) × 38.67 = HDL-C (mg/dL).

### Statistical analysis

Continuous variables were assessed for normality using the Shapiro-Wilk test and visual inspection of histograms. Data are presented as mean ± standard deviation for continuous variables, and as frequencies and percentages for categorical variables. Baseline characteristics were compared between METS-IR groups (split by the median value of 32) using the Wilcoxon rank-sum test for continuous variables and the chi-square test or Fisher’s exact test for categorical variables.

Univariate linear regression analyses were first performed to examine the associations between each candidate predictor (sex, age, tumor type, histology, bone metastasis, prior fracture, smoking, glucocorticoid use, alcohol consumption, and METS-IR) and each skeletal outcome (lumbar spine BMD, lumbar spine TBS, femoral neck BMD, and total hip BMD). To identify independent predictors, variables with a P-value < 0.10 in univariate analyses were entered into multivariable linear regression models for each outcome. Adjustment variables were selected based on a combination of statistical significance and clinical relevance. All statistical analyses were performed using R software (version 4.2.2; R Foundation for Statistical Computing, Vienna, Austria), and Free Statistics data analysis platform (version 2.3; Beijing Free Clinical Medical Technology Co., Ltd., Beijing, China). A two-tailed P-value < 0.05 was considered statistically significant.

Missing data were assessed for all variables. No imputation was performed. Complete-case analysis was used for each regression model; therefore, the number of participants included in each multivariable model differed slightly according to the completeness of the covariates included. Specifically, bone metastasis status was missing for 1 participant, and lumbar spine TBS could not be reliably computed for 2 participants owing to severe vertebral structural artifacts or surgical hardware. Consequently, multivariable models that included bone metastasis as a covariate were based on 126 participants, and all TBS-related analyses were based on 125 participants. Models without these variables included all 127 participants.

### Sample size estimation

The minimum sample size was calculated manually according to the commonly used rule that, for multivariable linear regression, the sample size should be at least 10 times the number of independent variables (≥10 participants per independent variable), which is the continuous-outcome analogue of the events-per-variable (EPV ≥10) rule (14). With a maximum of 10 candidate predictors planned for the adjusted models, the minimum required sample size was 100 participants. Allowing for approximately 10% missing or incomplete data, the target minimum sample size was set at 112 participants. The present study included 127 participants, which exceeded this prespecified minimum requirement.

## Results

### Patient selection

**Figure 1:**
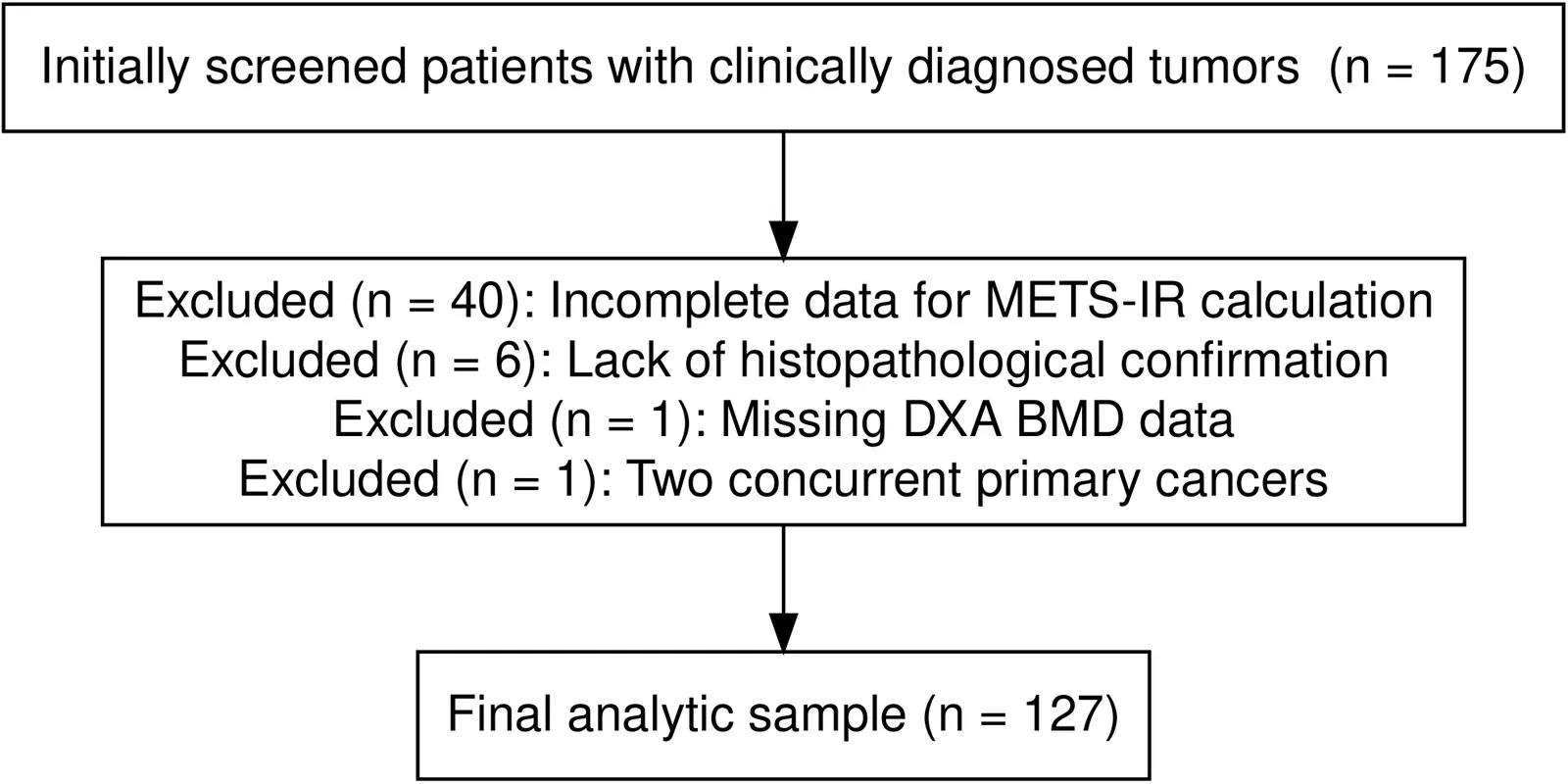
Flow chart illustrating the selection of the study population.

A total of 175 patients with clinically diagnosed tumors were initially screened. After applying the exclusion criteria, 6 patients were excluded for lack of histopathological confirmation of malignancy, 1 patient for missing dual-energy X-ray absorptiometry (DXA) bone mineral density (BMD) data, 1 patient for two concurrent primary cancers, and 40 patients for incomplete data required to calculate the Metabolic Score for Insulin Resistance (METS-IR). The remaining 127 patients constituted the final analytic sample (**Figure 1**).

### Baseline characteristics

The baseline demographics and clinical characteristics of the cohort, stratified by the median METS-IR value (32), are presented in **Table 1**. The median age was 63 years, and 57 patients (44.9%) were female. The most frequent tumor types were thoracic tumors (37.8%), abdominopelvic tumors (32.3%), head and neck tumors (15.0%), and lymphomas (15.0%). Adenocarcinoma was the predominant histological subtype (47.2%). Bone metastasis was present in 24 patients (18.9%), and a history of prior fracture was reported in 12 patients (9.4%). No statistically significant differences were observed between the low and high METS-IR groups with respect to age, sex distribution, tumor type, histological subtype, presence of bone metastasis, prior fracture history, smoking status, long-term glucocorticoid use, or alcohol consumption (all P > 0.05).

**Table 1:** Baseline demographic and clinical characteristics of cancer patients stratified by METS-IR group.

Characteristic	METS IR < 32 group (N = 63)	METS IR ≥ 32 group (N = 64)	p-value
Age (Mean ± SD)	61 ± 12	62 ± 9	0.785
Sex, n (%)			0.649
Female	27/63 (42.9%)	30 (46.9%)	
Male	36/63 (57.1%)	34 (53.1%)	
Tumor site, n (%)			0.252
Head and neck	7/63 (11.1%)	12 (18.8%)	
Lymphoma	8/63 (12.7%)	11 (17.2%)	
Thoracic	29/63 (46.0%)	19 (29.7%)	
Abdominopelvic	19/63 (30.2%)	22 (34.4%)	
Histological type, n (%)			0.081
Lymphoma	8/63 (12.7%)	11/64 (17.2%)	
Adenocarcinoma	37/63 (58.7%)	23/64 (35.9%)	
Non-squamous non-adenocarcinoma	5/63 (7.9%)	8/64 (12.5%)	
Squamous cell carcinoma	13/63 (20.6%)	22/64 (34.4%)	
Bone metastasis, n (%)	13/62 (21.0%)	11/64 (17.2%)	0.589
Prior fracture, n (%)	6/63 (9.8%)	6/64 (9.4%)	0.930
Smoking, n (%)	16/63 (25.4%)	14/64 (22.2%)	0.676
Long-term glucocorticoid use, n (%)	1/63 (1.6%)	0/64 (0.0%)	0.496
Alcohol, n (%)	6/63 (9.5%)	4/64 (6.3%)	0.530

METS-IR, Metabolic Score for Insulin Resistance; SD, standard deviation.

### Association of METS-IR with lumbar spine BMD, femoral neck BMD, total hip BMD, and lumbar spine TBS

BMD and TBS values according to METS-IR group are shown in **Table 2**. In univariate linear regression, male sex, younger age, presence of bone metastasis, and METS-IR met the pre-defined threshold (P < 0.10) and were therefore included in the multivariable model for lumbar spine BMD. After mutual adjustment, METS-IR remained independently and positively associated with lumbar spine BMD (β = 0.006 per unit increase, 95% CI 0.002 to 0.011, P = 0.007) (**Table 3**). Male sex, younger age, and the presence of bone metastasis were also independent predictors of higher lumbar spine BMD.

**Table 2:** Bone mineral density and trabecular bone score by METS-IR group.

Characteristic	METS IR < 32 group (N = 63)	METS IR ≥ 32 group (N = 64)	p-value
Lumbar spine BMD (Mean ± SD)	0.99 ± 0.16	1.05 ± 0.20	0.113
Lumbar spine TBS (Mean ± SD)	1.33 ± 0.07	1.35 ± 0.09	0.678
Total hip BMD (Mean ± SD)	0.82 ± 0.11	0.89 ± 0.17	0.027
Femoral neck BMD (Mean ± SD)	0.78 ± 0.10	0.85 ± 0.18	0.012

BMD, bone mineral density; TBS, trabecular bone score; METS-IR, Metabolic Score for Insulin Resistance; SD, standard deviation.

**Table 3:** Univariate and multivariate linear regression analysis of factors associated with lumbar spine BMD.

Characteristic	Univariable				Multivariable			
	N	Beta	95% CI	p-value	N	Beta	95% CI	p-value
Sex								
Female	57	/	/		57	/	/	
Male	70	0.062	0.002, 0.127	0.059	69	0.084	0.023, 0.145	0.008
Age	127	-0.003	-0.006, 0.000	0.028	126	-0.005	-0.008, -0.002	<0.001
Tumor site								
Head and neck	19	/	/					
Lymphoma	19	-0.030	-0.148, 0.089	0.624				
Thoracic	48	-0.049	-0.148, 0.050	0.334				
Abdominopelvic	41	-0.059	-0.160, 0.043	0.261				
Histological type								
Lymphoma	19	/	/					
Adenocarcinoma	60	-0.005	-0.101, 0.092	0.925				
Non-squamous non-adenocarcinoma	13	-0.013	-0.146, 0.119	0.842				
Squamous cell carcinoma	35	-0.031	-0.136, 0.074	0.561				
Bone metastasis								
No	102	/	/		102	/	/	
Yes	24	0.082	0.002, 0.162	0.048	24	0.107	0.031, 0.183	0.007
Prior fracture								
No	113	/	/					
Yes	12	-0.025	-0.136, 0.085	0.655				
Smoking								
No	96	/	/					
Yes	30	0.007	-0.070, 0.083	0.866				
Long-term glucocorticoid use								
No	126	/	/					
Yes	1	0.238	-0.126, 0.602	0.203				
Alcohol								
No	117	/	/					
Yes	10	-0.027	-0.147, 0.094	0.666				
METS IR	127	0.004	-0.001, 0.009	0.100	126	0.006	0.002, 0.011	0.007

Multivariable model adjusted for sex, age, bone metastasis.

CI, confidence interval; METS-IR, Metabolic Score for Insulin Resistance; BMD, bone mineral density.

For femoral neck BMD, univariate analysis identified age and METS-IR as significant factors. In the multivariable model, both age and METS-IR (β = 0.007 per unit, 95% CI 0.003 to 0.011, P < 0.001) were independently associated with femoral neck BMD (**Table 4**).

**Table 4:** Univariate and multivariate linear regression analysis of factors associated with femoral neck BMD.

Characteristic	Univariable				Multivariable			
	N	Beta	95% CI	p-value	N	Beta	95% CI	p-value
Sex								
Female	57	/	/					
Male	70	0.018	-0.034, 0.070	0.503				
Age	127	-0.003	-0.005, 0.000	0.030	127	-0.003	-0.006, -0.001	0.010
Tumor site								
Head and neck	19	/	/					
Lymphoma	19	0.000	-0.095, 0.096	0.992				
Thoracic	48	-0.004	-0.084, 0.076	0.922				
Abdominopelvic	41	-0.022	-0.104, 0.060	0.606				
Histological type								
Lymphoma	19	/	/					
Adenocarcinoma	60	-0.003	-0.081, 0.075	0.940				
Non-squamous non-adenocarcinoma	13	0.002	-0.105, 0.108	0.976				
Squamous cell carcinoma	35	-0.028	-0.112, 0.056	0.518				
Bone metastasis								
No	102	/	/					
Yes	24	0.044	-0.022, 0.110	0.194				
Prior fracture								
No	113	/	/					
Yes	12	-0.001	-0.090, 0.087	0.978				
Smoking								
No	96	/	/					
Yes	30	0.007	-0.055, 0.068	0.829				
Long-term glucocorticoid use								
No	126	/	/					
Yes	1	0.048	-0.247, 0.343	0.751				
Alcohol								
No	117	/	/					
Yes	10	-0.020	-0.116, 0.077	0.691				
METS IR	127	0.006	0.002, 0.010	0.002	127	0.007	0.003, 0.011	<0.001

Multivariable model adjusted for age and METS-IR.

CI, confidence interval; BMD, bone mineral density; METS-IR, Metabolic Score for Insulin Resistance.

In univariate analysis of total hip BMD, male sex, bone metastasis, and METS-IR were entered into the multivariable model. After adjustment, METS-IR remained significantly and positively associated with total hip BMD (β = 0.007 per unit, 95% CI 0.003 to 0.010, P < 0.001) (**Table 5**). Bone metastasis also exhibited a significant independent association, whereas male sex did not reach statistical significance.

**Table 5:** Univariate and multivariate linear regression analysis of factors associated with total hip BMD.

Characteristic	Univariable				Multivariable			
	N	Beta	95% CI	p-value	N	Beta	95% CI	p-value
Sex								
Female	57	/	/		57	/	/	
Male	70	0.049	-0.002, 0.099	0.064	69	0.046	-0.002, 0.094	0.065
Age	127	-0.001	-0.004, 0.001	0.330				
Tumor site								
Head and neck	19	/	/					
Lymphoma	19	0.048	-0.046, 0.142	0.317				
Thoracic	48	0.020	-0.058, 0.098	0.621				
Abdominopelvic	41	-0.009	-0.089, 0.071	0.825				
Histological type								
Lymphoma	19	/	/					
Adenocarcinoma	60	-0.037	-0.113, 0.039	0.342				
Non-squamous non-adenocarcinoma	13	-0.050	-0.155, 0.054	0.344				
Squamous cell carcinoma	35	-0.049	-0.132, 0.033	0.245				
Bone metastasis								
No	102	/	/		102	/	/	
Yes	24	0.055	-0.009, 0.120	0.095	24	0.070	0.008, 0.131	0.029
Prior fracture								
No	113	/	/					
Yes	12	-0.035	-0.123, 0.052	0.433				
Smoking								
No	96	/	/					
Yes	30	0.014	-0.046, 0.075	0.645				
Long-term glucocorticoid use								
No	126	/	/					
Yes	1	0.186	-0.102, 0.474	0.209				
Alcohol								
No	117	/	/					
Yes	10	-0.011	-0.106, 0.084	0.823				
METS IR	127	0.006	0.002, 0.010	0.003	126	0.007	0.003, 0.010	<0.001

Multivariable model adjusted for sex, bone metastasis, and METS-IR.

CI, confidence interval; BMD, bone mineral density; METS-IR, Metabolic Score for Insulin Resistance.

With respect to lumbar spine TBS, METS-IR was not significantly associated in univariate analysis (β = 0.001, 95% CI −0.001 to 0.003, P = 0.374) and was therefore not included in the multivariable model. Instead, male sex, age, and tumor type were selected. In the final multivariable model, male sex and younger age were independently associated with higher lumbar spine TBS, whereas tumor type was not significantly associated with TBS after adjustment (**Table 6**).

**Table 6:** Univariate and multivariate linear regression analysis of factors associated with lumbar spine TBS.

Characteristic	Univariable				Multivariable			
	N	Beta	95% CI	p-value	N	Beta	95% CI	p-value
Sex								
Female	56	/	/		56	/	/	
Male	69	0.029	0.000, 0.057	0.049	69	0.040	0.012, 0.068	0.007
Age	125	-0.002	-0.004, -0.001	<0.001	125	-0.003	-0.004, -0.001	<0.001
Tumor site								
Head and neck	19	/	/		19	/	/	
Lymphoma	19	-0.020	-0.072, 0.031	0.447	19	-0.022	-0.070, 0.026	0.375
Thoracic	48	-0.034	-0.077, 0.009	0.124	48	-0.021	-0.062, 0.019	0.307
Abdominopelvic	39	-0.040	-0.084, 0.005	0.082	39	-0.025	-0.068, 0.017	0.244
Histological type								
Lymphoma	19	/	/					
Adenocarcinoma	58	-0.012	-0.054, 0.031	0.584				
Non-squamous non-adenocarcinoma	13	-0.024	-0.082, 0.034	0.415				
Squamous cell carcinoma	35	-0.002	-0.047, 0.044	0.949				
Bone metastasis								
No	101	/	/					
Yes	23	0.014	-0.023, 0.051	0.448				
Prior fracture								
No	111	/	/					
Yes	12	0.013	-0.035, 0.062	0.590				
Smoking								
No	95	/	/					
Yes	29	0.005	-0.029, 0.039	0.785				
Alcohol								
No	116	/	/					
Yes	9	0.013	-0.042, 0.068	0.649				
METS_IR	125	0.001	-0.001, 0.003	0.374				

Multivariable model adjusted for sex, age, and tumor site.

CI, confidence interval; TBS, trabecular bone score; METS-IR, Metabolic Score for Insulin Resistance.

## Discussion

In this cross-sectional study of adult cancer patients, we demonstrated that a higher Metabolic Score for Insulin Resistance (METS-IR) was independently and positively associated with higher bone mineral density (BMD) at the lumbar spine, femoral neck, and total hip, after adjustment for outcome-specific covariates (lumbar spine BMD: sex, age, and bone metastasis; femoral neck BMD: age; total hip BMD: sex and bone metastasis). In contrast, METS-IR showed no significant association with lumbar spine trabecular bone score (TBS), a measure of trabecular microarchitecture. These findings suggest that while insulin resistance (IR) as assessed by METS-IR may be linked to greater bone mass, it does not necessarily confer better bone quality, highlighting a potential discrepancy between bone quantity and bone microstructural integrity in cancer patients with metabolic disturbance.

Notably, the presence of bone metastasis was also independently associated with higher BMD at the lumbar spine and total hip. While seemingly counterintuitive, this observation likely reflects the inclusion of patients with osteoblastic bone metastases—commonly seen in prostate, breast, and lung cancers—which can artifactually increase DXA-derived BMD values owing to sclerosis (15). Alternatively, patients with bone metastases may have received more intensive bisphosphonate or denosumab therapy, which could raise BMD (16, 17); however, detailed treatment data were not available in this study. The detection of bone metastasis via imaging should always be considered when interpreting BMD results.

The most intriguing finding of the present study is the discordance between BMD and TBS with respect to their associations with METS-IR. While BMD was positively linked to METS-IR, TBS was not, suggesting that IR may increase bone quantity without improving—and perhaps impairing—bone quality. This phenomenon has been elegantly described in type 2 diabetes mellitus (T2DM), where patients frequently have normal or even elevated BMD yet experience a paradoxically increased fracture risk, in part due to compromised bone microarchitecture as captured by lower TBS (18, 19). The accumulation of advanced glycation end-products (AGEs) in the bone matrix, accelerated by hyperglycemia and oxidative stress, leads to non-enzymatic collagen cross-linking, which stiffens the collagen network and reduces its ability to absorb energy, thereby increasing brittleness (20, 21). Furthermore, visceral adiposity and IR are associated with chronic low-grade systemic inflammation, elevated pro-inflammatory cytokines such as tumor necrosis factor-α and interleukin-6, and altered adipokine secretion (decreased adiponectin and increased leptin), all of which may negatively influence bone remodeling and trabecular connectivity (22–24). In our study, METS-IR did not capture these detrimental effects directly, but its failure to correlate with TBS suggests that IR-related bone quality deterioration may offset the apparent anabolic benefits on BMD. Future studies incorporating dynamic bone histomorphometry or high-resolution peripheral quantitative CT are needed to clarify the structural basis of this discrepancy.

We also found that younger age was independently associated with higher lumbar spine BMD, femoral neck BMD, and TBS, whereas no independent association between age and total hip BMD was observed. These findings are largely consistent with the well-established age-dependent decline in bone mass and microarchitecture (25). Male sex was independently associated with higher lumbar spine BMD and TBS, but only marginally with hip BMD, perhaps reflecting sex-specific patterns of cancellous versus cortical bone loss (25). Tumor type was not an independent predictor of TBS after adjustment for age and sex, implying that the observed associations are driven more by systemic metabolic factors than by cancer histology.

The clinical implications of our findings are noteworthy. METS-IR is a simple, non-insulin-based index that can be readily calculated from routine laboratory and anthropometric data, making it a pragmatic tool for large-scale or retrospective studies in oncology settings where insulin measurements are often unavailable. The positive BMD-METS-IR relationship could help identify patients with low bone mass who might benefit from early intervention. However, the absence of a corresponding beneficial association with TBS urges caution: a normal or even high BMD in an insulin-resistant cancer patient may mask underlying trabecular deterioration. Clinicians should therefore consider incorporating TBS or other assessments of bone quality when evaluating fracture risk in metabolically compromised cancer patients.

Several limitations of this study must be acknowledged. First, its cross-sectional design precludes causal inference; longitudinal studies are required to determine whether changes in METS-IR over time predict bone loss or incident fractures. Second, the sample size, although comparable to other single-center studies in this field, limited the power to detect small effect sizes and to perform extensive subgroup analyses by sex or specific cancer type. Third, we lacked data on insulin levels, glycated hemoglobin, bone turnover markers, and adipokines, which could have provided mechanistic insights into the IR-bone relationship. Fourth, detailed information on cancer treatment—such as chemotherapy regimens, endocrine therapy, glucocorticoid exposure, and bone-modifying agent use—was not available and could influence both metabolic status and bone health. Fifth, TBS measurements can be confounded by excessive abdominal fat or severe degenerative changes, although our scans were centrally reviewed to minimize this. Sixth, our study population was heterogeneous in terms of tumor types; while this enhances generalizability, it also introduces variability that may obscure cancer-specific effects. Despite these limitations, the present study fills an important gap in the literature and offers clinically relevant insights. Although the current cross-sectional analysis cannot establish causality, the consistent and independent associations observed across multiple skeletal sites provide a robust foundation for future longitudinal research and highlight the relevance of integrating metabolic and skeletal assessments in cancer care.

In conclusion, this study demonstrates that METS-IR is independently associated with higher BMD at multiple skeletal sites in cancer patients, but not with better trabecular microarchitecture as assessed by TBS. The divergence between bone quantity and bone quality in the setting of insulin resistance may have important implications for fracture risk assessment in this population. Future prospective studies are warranted to validate these findings and to explore the longitudinal impact of metabolic health on skeletal integrity in oncology patients.

## Data Availability

The raw data supporting the conclusions of this article will be made available by the authors, without undue reservation.

## References

[1] Brufsky AM . Cancer treatment-induced bone loss: pathophysiology and clinical perspectives. Oncologist. (2008) 13:187–95. doi: 10.1634/theoncologist.2007-0152 18305064

[2] Coleman R Body JJ Aapro M Hadji P Herrstedt J ESMO Guidelines Working Group . Bone health in cancer patients: ESMO Clinical Practice Guidelines. Ann Oncol. (2014) 25:iii124–137. doi: 10.1093/annonc/mdu103 24782453

[3] Hong AR Kim JH Lee KH Kim TY Im SA Kim TY . Long-term effect of aromatase inhibitors on bone microarchitecture and macroarchitecture in non-osteoporotic postmenopausal women with breast cancer. Osteoporos Int. (2017) 28:1413–22. doi: 10.1007/s00198-016-3899-6 28083668

[4] Guise TA . Bone loss and fracture risk associated with cancer therapy. Oncologist. (2006) 11:1121–31. doi: 10.1634/theoncologist.11-10-1121 17110632

[5] Link TM Kazakia G . Update on imaging-based measurement of bone mineral density and quality. Curr Rheumatol Rep. (2020) 22:13. doi: 10.1007/s11926-020-00892-w 32270332 PMC7875476

[6] Harvey NC Glüer CC Binkley N McCloskey EV Brandi M-L Cooper C . Trabecular bone score (TBS) as a new complementary approach for osteoporosis evaluation in clinical practice. Bone. (2015) 78:216–24. doi: 10.1016/j.bone.2015.05.016 25988660 PMC4538791

[7] Ran B Wei F Gong J Xu H . Application and prospect of trabecular bone score in differentiated thyroid cancer patients receiving thyrotropin suppression therapy. Front Endocrinol. (2022) 13. doi: 10.3389/fendo.2022.1004962 36313757 PMC9596913

[8] Armutcu F McCloskey E . Insulin resistance, bone health, and fracture risk. Osteoporos Int. (2024) 35:1909–17. doi: 10.1007/s00198-024-07227-w 39264439

[9] Hung Y-T Yu T-H Alizargar J . Insulin resistance and bone mineral density: A comprehensive examination using UK biobank data. Healthcare. (2024) 12:2502. doi: 10.3390/healthcare12242502 39765929 PMC11727659

[10] Shirinezhad A Azarboo A Ghaseminejad-Raeini A Kanaani Nejad F Zareshahi N Amiri SM . A systematic review of the association between insulin resistance surrogate indices and bone mineral density. Front Endocrinol. (2024) 15. doi: 10.3389/fendo.2024.1499479 39744182 PMC11688183

[11] Campillo-Sánchez F Usategui-Martín R Ruiz-de Temiño Á Gil J Ruiz-Mambrilla M Fernández-Gómez JM . Relationship between insulin resistance (HOMA-IR), trabecular bone score (TBS), and three-dimensional dual-energy X-ray absorptiometry (3D-DXA) in non-diabetic postmenopausal women. J Clin Med. (2020) 9:1732. doi: 10.3390/jcm9061732 32503328 PMC7355807

[12] Bello-Chavolla OY Almeda-Valdes P Gomez-Velasco D Viveros-Ruiz T Cruz-Bautista I Romo-Romo A . METS-IR, a novel score to evaluate insulin sensitivity, is predictive of visceral adiposity and incident type 2 diabetes. Eur J Endocrinol. (2018) 178:533–44. doi: 10.1530/EJE-17-0883 29535168

[13] Pan L Zou H Meng X Li D Li W Chen X . Predictive values of metabolic score for insulin resistance on risk of major adverse cardiovascular events and comparison with other insulin resistance indices among Chinese with and without diabetes mellitus: Results from the 4C cohort study. J Diabetes Invest. (2023) 14:961–72. doi: 10.1111/jdi.14024 37132055 PMC10360377

[14] Peduzzi P Concato J Kemper E Holford TR Feinstein AR . A simulation study of the number of events per variable in logistic regression analysis. J Clin Epidemiol. (1996) 49:1373–9. doi: 10.1016/s0895-4356(96)00236-3 8970487

[15] Martineau P Morgan SL Leslie WD . Bone mineral densitometry reporting: pearls and pitfalls. Can Assoc Radiol J. (2021) 72:490–504. doi: 10.1177/0846537120919627 32309998

[16] Baldessari C Pipitone S Molinaro E Cerma K Fanelli M Nasso C . Bone metastases and health in prostate cancer: from pathophysiology to clinical implications. Cancers (Basel). (2023) 15:1518. doi: 10.3390/cancers15051518 36900309 PMC10000416

[17] D’Oronzo S Cives M Lauricella E Stucci S Centonza A Gentile M . Assessment of bone turnover markers and DXA parameters to predict bone metastasis progression during zoledronate treatment: a single-center experience. Clin Exp Med. (2024) 24:7. doi: 10.1007/s10238-023-01280-1 38240866 PMC10798926

[18] Bhattacharya S Nagendra L Chandran M Kapoor N Patil P Dutta D . Trabecular bone score in adults with type 1 diabetes: a meta-analysis. Osteoporos Int. (2024) 35:105–15. doi: 10.1007/s00198-023-06935-z 37819402

[19] Trandafir A-I Sima O-C Gheorghe A-M Ciuche A Cucu A-P Nistor C . Trabecular bone score (TBS) in individuals with type 2 diabetes mellitus: an updated review. J Clin Med. (2023) 12:7399. doi: 10.3390/jcm12237399 38068450 PMC10707110

[20] Vashishth D Dhaliwal R Rubin M . AGEs (Advanced Glycation End-products) in bone come of age. Bone. (2025) 190:117301. doi: 10.1016/j.bone.2024.117301 39442854 PMC13016710

[21] Wang B Vashishth D . Advanced glycation and glycoxidation end products in bone. Bone. (2023) 176:116880. doi: 10.1016/j.bone.2023.116880 37579812 PMC10529863

[22] Tilg H Ianiro G Gasbarrini A Adolph TE . Adipokines: masterminds of metabolic inflammation. Nat Rev Immunol. (2025) 25:250–65. doi: 10.1038/s41577-024-01103-8 39511425

[23] Ren Y Zhao H Yin C Lan X Wu L Du X . Adipokines, hepatokines and myokines: focus on their role and molecular mechanisms in adipose tissue inflammation. Front Endocrinol. (2022) 13. doi: 10.3389/fendo.2022.873699 35909571 PMC9329830

[24] Engin A . Reappraisal of adipose tissue inflammation in obesity. Adv Exp Med Biol. (2024) 1460:297–327. doi: 10.1007/978-3-031-63657-8_10 39287856

[25] Shanbhogue VV Brixen K Hansen S . Age- and sex-related changes in bone microarchitecture and estimated strength: A three-year prospective study using HRpQCT. J Bone Miner Res. (2016) 31:1541–9. doi: 10.1002/jbmr.2817 26896351

